# Training, supervision and quality of care in selected integrated community case management (iCCM) programmes: A scoping review of programmatic evidence

**DOI:** 10.7189/jogh.04.020403

**Published:** 2014-12

**Authors:** Xavier Bosch–Capblanch, Claudine Marceau

**Affiliations:** 1 Swiss Tropical and Public Health Institute, Basel, Switzerland; 2 University of Basel, Basel, Switzerland; 3 Freelance consultant, attached to the Swiss Tropical and Public Health Institute, Basel, Switzerland

## Abstract

**Aim:**

To describe the training, supervision and quality of care components of integrated Community Case Management (iCCM) programmes and to draw lessons learned from existing evaluations of those programmes.

**Methods:**

Scoping review of reports from 29 selected iCCM programmes purposively provided by stakeholders containing any information relevant to understand quality of care issues.

**Results:**

The number of people reached by iCCM programmes varied from the tens of thousands to more than a million. All programmes aimed at improving access of vulnerable populations to health care, focusing on the main childhood illnesses, managed by Community Health Workers (CHW), often selected bycommunities. Training and supervision were widely implemented, in different ways and intensities, and often complemented with tools (eg, guides, job aids), supplies, equipment and incentives. Quality of care was measured using many outcomes (eg, access or appropriate treatment). Overall, there seemed to be positive effects for those strategies that involved policy change, organisational change, standardisation of clinical practices and alignment with other programmes. Positive effects were mostly achieved in large multi–component programmes. Mild or no effects have been described on mortality reduction amongst the few programmes for which data on this outcome was available to us. Promising strategies included teaming–up of CHW, micro–franchising or social franchising. On–site training and supervision of CHW have been shown to improve clinical practices. Effects on caregivers seemed positive, with increases in knowledge, care seeking behaviour, or caregivers’ basic disease management. Evidence on iCCM is often of low quality, cannot relate specific interventions or the ways they are implemented with outcomes and lacks standardisation; this limits the capacity to identify promising strategies to improve quality of care.

**Conclusion:**

Large, multi–faceted, iCCM programmes, with strong components of training, supervision, which included additional support of equipment and supplies, seemed to improve selected quality of care outcomes. However, current evaluation and reporting practices need to be revised in a new research agenda to address the methodological challenges of iCCM evaluations.

It is widely recognized that there are effective interventions to prevent, detect, control and manage the most common diseases in poorly developed contexts, such as those affecting children in low– and middle–income countries [1]. However, it is equally acknowledged that the delivery of these interventions is severely hampered by rudimentary or decayed health systems, where essential dimensions of quality of care, such as availability, access and utilisation of services [2], are hardly fulfilled [3].

Innovative approaches do exist to address health care delivery faults, ultimately aiming at addressing quality of care shortcomings. The Integrated Community Case Management (iCCM) promoted by the World Health Organisation (WHO) / United Nations Children Fund (UNICEF) [4], encompasses a series of strategies and activities taking health care closer to communities. In this approach, Community Health Workers (CHW) typically serve as the first point of contact between communities and services.

As any other intervention or strategy, iCCM programmes have to be tested or evaluated in order to describe successes, failures and factors related to them. Rigorous research evidence on the effects of iCCM is scanty [5,6]. Furthermore, iCCM programmes often encompass multiple components which complicates their evaluations. Often, evidence on iCCM programmes has to come from programmatic documents supported by operational research of varying quality.

The aims of this article are to report on the components of selected iCCM programmes and to draw lessons learned from existing evaluations of those programmes, through a scoping literature review of programmatic documentation. We will not attempt to estimate or synthesise the effects of iCCM interventions in primary or secondary programmatic or health–related outcomes, but will provide illustrative examples.

## METHODS

A scoping, structured literature review of programmatic information evidence was carried out. ‘Structured review’ refers to a review of the literature which pragmatically adapts standard systematic reviews’ methodology, such as the one used for Cochrane Review [7], yet remains transparent in relation to its methods and rationale for adaptations. This review was based on documents provided by stakeholders since UNICEF defined the focus and scope of the review. Outcomes were only generically predefined as human resources and quality of care related outcomes.

Included documents referred to programmes reported by selected partners proposed by UNICEF. There were no restrictions based on the types of documents, types of studies or types of evidence within them. Quality of evidence was no formally assessed and therefore there were no exclusions based on this criterion. However, three levels of quality of evidence were defined to support the interpretation of findings on the effects of the programmes: low quality when the source of evidence was based on qualitative data or opinions; moderate quality when quantitative methods were used and described in the source documents; and high quality when findings were presented with some measure of statistical significance (‘+’. ‘++’ and ‘+++’, respectively).

Twenty nine programmes were proposed and provided by UNICEF and partners. Three types of data were extracted: (a) features describing the programmes (eg, name, funding, objectives, time frame); (b) programme tools highlighted as promising approaches to improve health care; (c) evidence on the effects of tools and approaches. This information was synthesised across programmes into two thematic areas: human resources and quality of care (only the latter is reported in this article).

Descriptions of iCCM programmes and their features are presented narratively and, where data are available, quantitative information is also included in the text or as tables. Due to the large variability in the amount and in the types and quality of evidence across iCCM programmes, no attempt has been made to carry out meta–analyses of quantitative estimates across iCCM programmes. References to particular iCCM programmes are made within brackets with the terms used in the documents and the country names as appropriate.

There was no overreaching quality of care framework across all programmes and authors accepted an estimate or an indicator to be related to quality of care if it referred to the events in the delivery of care (from availability of care to effective coverage) and health related outcomes. Since this is not a review on the effects of iCCM interventions, we have selected only some indicators best related to quality of care or serving as illustrative examples, from the very large amount of indicators reported in some programmes.

## RESULTS

### Overview of iCCM programmes’ objectives and strategies

A total of 29 iCCM programmes were included in this review. All programmes were implemented in African countries, but one, in Myanmar. **Table 1** lists the included programmes alongside the main implementing organisation, partners and programmes’ start and end years. The documentation scrutinised referred to programmes or phases ending between 2010 and 2013 (66% of programmes), two others were older (2005 and 2008), another one was ongoing (ending in 2015) and for the remaining seven dates were missing

**Table 1:** Programmes included in the review, partners and duration

Programme reference	Main organisation	Partners	Year start	Year end
CHW Backpack Plus	Frog	UNICEF; MDG; Save the Children	2013	2013
Concern Burundi	Concern; USAID;	MOH National Malaria Program (PNILP), WHO, UNICEF, the Global Fund, Pathfinder/MSH, and World Relief	2012	NA
Concern Niger	USAID; Concern	NA	NA	NA
Concern Rwanda KabehoMwana	USAID; Concern	International Rescue Committee; World Relief; Health Grants Program	2006	2011
CORE group	CORE	Plan; USAID; Save the children	NA	NA
CORE group – Cameroon	Plan	USAID; Child Survival and Health Grants Program (CSHGP)	2000	2008
CORE group – Malawi	World Relief	USAID	2000	2005
IRC Sierra Leone	International Rescue Committee	CIDA (funding)	2005	NA
Living Goods Uganda	Living Goods	BRAC	2006	2013
MC South Sudan	Malaria Consortium	UNICEF; WHO; PSI; Save the Children; IRC; Catholic Dioscese of Torit; BRAC	2010	2013
MC Uganda	Malaria Consortium	CIDA; MOH Uganda; UNICEF; WHO; ACCORDIA; Global Health Foundation; USAID	2010	2015
MOH Ethiopia	MOH Ethiopia	Johns Hopkins Bloomberg School of Public Health (IIP–JHU); iCCM evaluation: IIP–JHU, ABH Services, PLC	2011	2013
MOH Madagascar	MOH Madagascar	UNICEF; USAID/Santénet2 (SN2); Malaria National Strategic Application (NSA) Grant of the Global Fund for HIV/AIDS, TB and Malaria (GFATM)	2008	2013
MOH Malawi	MOH Malawi	Global Fund grant for scale–up; WHO/UNICEF Training material	2008	2011
MOH Mozambique	MOH Mozambique	Evaluation: UNICEF, USAID/TRAction, UEM and JHSPH. UNICEF, WHO, USAID, Save the Children and Malaria Consortium.	2012	2013
MOH Uganda	MOH Uganda	UNICEF; WHO; USAID	NA	2010
PSI Cameroon CIDA	CIDA; PSI	CIDA	2009	2013
PSI DRC CIDA	CIDA; PSI	NA	2009	2013
PSI Malawi CIDA	CIDA; PSI	2 other partners	2009	2013
PSI Mali CIDA	CIDA; PSI	NA	2009	2013
PSI Madagascar	PSI	NA	NA	2011
PSI Myanmar SPH Franchise	PSI/Myanmar	Global Health Group	2008	2010
PSI South Sudan	PSI	Global Fund and CIDA; IRC; Save the Children; Malaria Consortium	2009	2013
PSI Uganda Five & Alive Franchise	PSI	PACE	2010	2013
Save Malawi	Save the Children	CIDA; MOH; (for study: JHU, NSO, Save the Children); (for medicine: CIDA, Everyone campaign, Bank of America)	2009	2012
Save Mozambique	Save the Children	CIDA; INE Mozambique	2010	2012
Save South Sudan	Save the Children	Global Fund & CIDA	2009	2013
Save Zambia	Save the Children	NA	2008	2012
USAID BASICS DRC (tools only)	USAID/BASICS, the DRC, MOH	UNICEF, WHO, GTZ, IRC and MSH	NA	NA
IRC Ethiopia	IRC	NA	NA	NA
Last Mile Health Liberia	Tyatien Health	NA	NA	NA

CHW – community health worker, BRAC – Bangladesh Rural Advancement Committee, CIDA – Canadian International Development Agency, DRC – Democratic Republic Congo, GTZ – Deutsche Gesellschaft für Technische Zusammenarbeit, INE – Instituto Nacional de Estadística, IRC – International Red Cross, MC – Malaria Consortium, MDG – Millennium Development Goals, MOH – Ministry of Health, MSH – Management Sciences for Health, NSO – National Statistics Office, PNILP – Programme National Intégré de Lutte contre le Paludisme, PSI – Population Services International, SPH – Sun Primary Health, UEM – Universidade Eduardo Mondlane, NA – not applicable

All programmes shared a common objective, which had to do with increasing access to good quality health services by poor populations, with a special focus on infants’ and children’s diseases, through the deployment of CHW, implementing Integrated Management of Childhood Illnesses (IMCI). 11 (40% of the 28 programmes with this information) included in their objectives morbidity and mortality targets (eg, ‘Backpack plus’, ‘Concern Niger’).

Basic clinical care was often complemented with other strategies (eg, ‘Backpack plus’, ‘MC Sudan South’), such as policy influence or advocacy (eg, ‘CORE–group’), health systems strengthening (eg, ‘Concern Niger’), provision of supplies and supply management (eg, ‘MC Uganda’), good clinical practices (eg, ‘MC Uganda’) or improve data transmission (eg, ‘Sizika’). The most common conditions addressed were, by far: malaria (or fever), diarrhoea and pneumonia (or respiratory symptoms), in children.

Programmes were often rooted in the communities themselves, but there were also examples where at least some services were integrated into the formal health sector at primary health care level (eg, ‘MOH Ethiopia’, ‘MOH Mozambique’, ‘PSI Uganda’).

The size of the programmes in terms of the number of population reached varied across programmes, phases within the same programme, population counted (ie, whole population or children of different age groups) and ways of measuring it (ie, as population in the catchment area or as population effectively treated). Median population (sometimes whole population, sometimes under–fives) was 304 245 (interquartile range: 108 484 to 536 616), ranging from a few thousands (eg, ‘Concern Burundi’ 37 379 children; ‘CORE–Cameroon’ 38 009 children) to the hundreds of thousands (‘Save South Sudan’ 125 035 children; ‘PSI–Cameroon’ 372 460 children; ‘Concern Burundi’ 310 129 vulnerable women; ‘PSI Malawi’ 304 245 children; ‘IRC Sierra Leone’ 605 981 population; ‘PSI DRC’ 636 000 population; ‘PSI South Sudan’ 722 708 population, ‘Save Mozambique’ 953 959 population); and beyond the million in ‘Save Malawi’ (1 435 219 population) and ‘PSI Malawi’ (2 336 255 population). In 14 programmes this piece of data was not retrievable. It is worth noting that studies evaluating iCCM strategies often used sub–samples of the covered population.

### Community health workers

CHW are at the core of iCCM. They are designated in different ways depending on the iCCM programme (**Table 2**). Names are in part descriptive of the functions CHW carry out but also respond to the names that may have been used in the past in certain countries (eg, Health Extension Workers in Ethiopian programmes). For the sake of clarity and simplification, we use the generic term CHW in this article.

**Table 2:** Designation of community health workers (CHW) as documented in the programmes

Programme	Designation of CHW
CHW Backpack Plus	Community Health Worker
Concern Burundi	Community Health Worker Agents de Santé Communtaire
Concern Niger	Community Health Worker
Concern Rwanda KabehoMwana	Community health workers; Community Based Distributors
CORE group – Cameroon	Community Health Worker
CORE group – Malawi	Health Surveillance Associates
IRC Sierra Leone	Community Based Distributors
Living Goods Uganda	Sales Representatives or Health Promoters
MC South Sudan	Community Drug Distributors; Community Based Distributor ; Community Health Workers
MC Uganda	Village Health Team
MOH Ethiopia	Health Extension Workers
MOH Madagascar	Community Health Volunteers; Agents Communautaires
MOH Malawi	Health Surveillance Associates; Community Health Based Workers;
MOH Mozambique	Community Health Workers; Agente Polivalente Elementar; Traditional Birth Attendants
MOH Uganda	Village Health Team members
PSI Cameroon CIDA	Community relais
PSI DRC CIDA	Community relais
PSI Malawi CIDA	Health Survelliance Agents
PSI Mali CIDA	Community Relais
PSI Madagascar	Agent de Santé Communitaire
PSI Myanmar SPH Franchise	Sun Primary Health
PSI South Sudan	Community Based Distributors; front line workers; Home Health Promoters; Community Health Workers
PSI Uganda Five & Alive Franchise	Community–based Village Health Team
Save Malawi	Health Surveillance Assistants
Save Mozambique	Agente Polivalente Elementar
Save South Sudan	CBDs = Community Based Distributors
Save Zambia	Community Health Workers
USAID BASICS DRC (tools only)	Community Health Workers
IRC Ethiopia	Health Extension Workers

CIDA – Canadian International Development Agency, DRC – Democratic Republic Congo; IRC – International Red Cross, MC – Malaria Consortium, MOH – Ministry of Health, PSI – Population Services International, SPH – Sun Primary Health

Activities carried out in the programmes (mainly by CHW) could fall into two main groups as identified in the programmes documents:

1) provision of clinical care (with or without other components, such as health promotion and prevention);

2) provision of supplies, mainly medical supplies (eg, drugs), through social franchising schemes (eg, ‘PSI Myanmar’, ‘PSI Uganda – Five & Alive’, ‘Living goods Uganda’) or using regular procurement schemes

(Social franchising is the provision of affordable services by the non–profit health sector, complying with franchise standards targeting underserved communities [8]; micro–franchising refers to small scale entrepreneurship by CHW [9]).

Treatment conditions included: malaria, diarrhoea and respiratory diseases assessment and early treatment, conjunctivitis, malnutrition, new–born at risk, ear infections, sexually transmitted infections and HIV testing.

Health promotion and disease prevention focused on malaria, diarrhoea and respiratory diseases recognition and health seeking behaviours, immunisation, nutrition, water and sanitation, maternal and new–born care, reproductive health and family planning, breastfeeding, complementary feeding, insecticide treated nets, malaria preventive treatment, TB prevention and treatment.

CHW were selected using a wide range of different criteria. For example, the main cadres selected in ‘CHW Backpack plus’ were supervisors of primary care facilities. Some CHW had basic education (O–level graduates in ‘CORE – Malawi’), or a minimum of five years of formal education (‘MOH Madagascar’), grade 10 junior certificate (‘MOH Malawi’, ‘Save Malawi’), minimal literacy with basic numerical competence (‘MOH Mozambique’, ‘PSI Cameroon CIDA’, ‘PSI DRC CIDA’, ‘Save Mozambique’) or even it might not be required any level of literacy (‘IRC Sierra Leone’).Occasionally, eligibility criteria could include already being a CHW, Traditional Birth Attendant (TBA), drug distributors or alike, in order to become member of Villages Health Teams (‘MOH Uganda’). In ‘Concern Rwanda KabehoMwana’ CHW selected themselves a cell coordinator. More rarely, CHW were staff from the MOH (eg, PSI Malawi CIDA, where the lowest rank of MOH employees are eligible).

CHW were selected by community members (10 programmes), community leaders (4 programmes), by the MOH (2 programmes), or by governments (1 programme) or NGOs (1 programme). In detail, CHW were selected by: MOH (‘MOH Malawi’), communities (Community Based Distributors in ‘IRC Sierra Leone’; ‘MC South Sudan’; ‘Save South Sudan’; by popular vote in ‘MOH Uganda’), both (‘PSI Cameroon CIDA’; in this case, though, the choice was made by their peers; ‘Save Mozambique’), government (‘Save Malawi’), or MOH and a local NGO (‘PSI DRC CIDA’, the final selection being made by the head nurse in collaboration with community leaders). In ‘MOH Madagascar’ most CHW were selected by their communities with some involvement of the traditional chieftaincy or the Community Health Committee. Involvement of villagers and chieftaincy was also reported in ‘PSI Madagascar’ and in ‘PSI South Sudan’. In ‘PSI Myanmar SPH Franchise’ CHW were recruited among auxiliary midwives, already existing CHW, farmers, or from other areas of activity. Additional criteria included being residents in the villages they were meant to serve (‘PSI Cameroon CIDA’, ‘PSI DRC CIDA’, ‘Save Malawi’, ‘Save Mozambique’) or being married (‘PSI DRC CIDA’). Interestingly, there was some information on exclusion criteria (ie, candidates who were NOT eligible); eg, political leaders or those imposed by political leadership (‘MOH Uganda’).

The number of CHW involved was difficult to assess because depended on the time–span of the programme, the degree of scaling up and the different types of health care workers reached. **Table 3** shows the approximate number of CHW involved in the programmes, when available in the source documents (median 1441, interquartile range 732 to 2582).

**Table 3:** Number of community health workers (CHW) involved*

Programme	Number of CHW
Concern Burundi	317
Concern Rwanda KabehoMwana	6100
CORE group – Malawi	2400 to 3060
IRC Sierra Leone	12 000
Living Goods Uganda	50 per district
MC South Sudan	715 to 1683
MC Uganda	5800 Village Health Teams, 800 CHW
MOH Ethiopia	137 under study; total 35 000
MOH Madagascar	4800
MOH Malawi	2709 to 10 000
MOH Mozambique	240
MOH Uganda	5 per village
PSI Cameroon CIDA	2454
PSI DRC CIDA	748
PSI Malawi CIDA	1639
PSI Mali CIDA	1936
PSI Myanmar SPH Franchise	1169
PSI South Sudan	1283
Save Malawi	838
Save Mozambique	273
Save South Sudan	1474
IRC Ethiopia	671

CIDA – Canadian International Development Agency, DRC – Democratic Republic Congo; IRC – International Red Cross, MC – Malaria Consortium, MOH – Ministry of Health, PSI – Population Services International, SPH – Sun Primary Health

*Not all programmes reported information on this area.

Disaggregation by CHW gender was only possible for a few programmes. For example, in ‘IRC Sierra Leone’ (26% females and 74% males of 2207 trained CHW), ‘MOH Ethiopia’ and ‘Save Mozambique’ (all females in both programmes, 137 and 273, respectively), ‘MOH Madagascar’ (half females and half males, of 4800), ‘PSI DRC CIDA’ (4% females and 96% males of 748 CHW), and in ‘Save Malawi’ (25% females and 75% males of 838 CHW).

Programme documents described several types of incentives. The majority of incentives were goods and even work equipment and tools (9 of the 17 programmes with data, 53%). Only in Malawi did CHW receive proper salaries. Incentives also included intangibles such as recognition and reputation. In detail:

• ‘incentives architecture’, similar to a career path (‘CHW Backpack plus’);

• reputation and recognition: ‘MOH Madagascar’, ‘PSI DRC CIDA’; increase in client flow using services (‘PSI Uganda Five & Alive Franchise’);

• performance–based financing mechanisms (‘Concern Rwanda KabehoMwana’, ‘PSI Myanmar SPH Franchise’);

• goods: soaps and batteries (‘IRC Sierra Leone’); T–shirt (‘Living Goods Uganda’); cap, T–shirt, torch, jerry cans, certificates, soap (‘MC Sudan South’); bicycles and T–shirts (‘MC Uganda’); bicycles, uniforms, T–shirts (‘PSI Malawi CIDA’); cellphone (‘PSI Mali CIDA’); sugar, salt, soap, bicycles, gumboots, clear bags and rain jackets (‘PSI South Sudan’); cycles, stainless steel spoons, medicine cups, water cups, plastic medicine bags, basins and a water container (‘Save Malawi’); soap, jugs, spoons, jerry cans, pair of scissors, medicine bag, pens, lunch and transport during refresher trainings (‘Save South Sudan’);

• percentage of benefits: loan start–up bag with US$ 60 worth of merchandise and 15% to 20% percent of whatever they sell (‘Living Goods Uganda’);

• per diem and allowances: for training (‘MOH Madagascar’); for reporting (‘PSI Cameroon CIDA’); travel allowance (‘PSI DRC CIDA’);

• cash: 1575 to 3150 South–Sudan pounds for staff; ‘MOH Madagascar’ (some CHW); ‘PSI South Sudan’; ‘Save Mozambique’;

• salaries: ‘MOH Malawi’, ‘Save Malawi’.

Besides, in some programmes, the training itself (‘PSI Malawi CIDA’) or certificates (‘PSI Madagascar’), supervision (‘PSI DRC CIDA’), supplies and working tools were mentioned as motivators or incentives to improve CHW performance (‘MOH Uganda’, ‘PSI Madagascar’).

### Training and supervision

Training schedules, length and approaches varied greatly across programmes. **Table 4** details the length of training for those programmes which had this information available. Median training length was 2 weeks (interquartile range from 6 to 43 days), depending on the contents and competences to be achieved.

**Table 4:** Duration of training of community health workers (CHW)*

Programme	Training duration
Concern Burundi	3 weeks
IRC Sierra Leone	6 days
Living Goods Uganda	4 weeks
MC South Sudan	6 days
MC Uganda	5 days
MOH Malawi	10 weeks
MOH Uganda	6 days
MOH Ethiopia	1 year
MOH Madagascar	8 months
PSI Cameroon CIDA	3 days
CORE group – Malawi	8 weeks
PSI DRC CIDA	From 2 to 3 days (depending on type of CHW)
PSI Malawi CIDA	6 days to 10 weeks (depending on competences)
PSI South Sudan	6 days
Save Malawi	6 days to 12 weeks (depending on competences)
Save Mozambique	6 days to 4 months
Save South Sudan	7 days
USAID BASICS DRC	6 days

CIDA – Canadian International Development Agency, DRC – Democratic Republic Congo; IRC – International Red Cross, MC – Malaria Consortium, MOH – Ministry of Health, PSI – Population Services International

*Not all programme reported information on this area.

Training was mainly formal in 8 out of 15 programmes (53%) with information on this area. Other approaches were present in 1 or 2 programmes as detailed below: formal training courses or refreshments (eg, annual in Concern Rwanda KabehoMwana and IRC Sierra Leone; biannual in ‘PSI Cameroon CIDA’; monthly in ‘PSI DRC CIDA’; ‘MC Uganda’, ‘Save Mozambique’, ‘MOH Ethiopia’ or ‘Save South Sudan’), mentorship programmes (‘MOH Madagascar’), including both theory and practical on–the–job training (e,g. ‘CHW Backpack plus’). Other approaches included (‘Concern Burundi’): interactive lessons where CHW learned to fill the tools and used their experience, exercises, demonstrations and role plays; similarly in ‘MC South Sudan’, ‘PSI DRC CIDA’; or even practical cases in in–patient health facilities (‘MOH Malawi’, ‘Save Malawi’). Only in ‘PSI DRC CIDA’ and ‘PSI Mali CIDA’ documentation it was mentioned that training was conducted using local languages as well.

Training focused on CHW but included other personnel as well (eg, health facility staff and district–level officials in ‘Concern Niger’; caregivers in individual households in ‘CORE–Malawi’); and might have the active involvement of supervisors in the training (USAID BASICS DRC). A cascade training approach was implemented in ‘Concern Burundi’ where MOH staff trained programme staff and the District Health Team, and then those trained CHW and health centre staff. In ‘MC Sudan South’, ‘MOH Uganda’ and ‘PSI DRC CIDA’ a cascade training was also implemented.

The contents of the training were mainly around clinical care; for example: to assess, classify, refer or treat sick children; to counsel the caretaker on home management follow up; to recognize and treat sick children aged 2 to 59 months with fever, diarrhoea, and pneumonia; to refer children to health facilities if they were less than 2 months, if they presented with illnesses other than fever, diarrhoea or pneumonia or if they showed any “danger signs”; if there were stock–outs, or if after treatment, the child’s condition failed to improve or worsened (IRC Sierra Leone). Similar skills were targeted in other programmes. Training included also the use of equipment (eg, respiratory timers in ‘MC Uganda’), Behaviour Change Communication (‘Concern Rwanda KabehoMwana’), reproductive health and family planning (‘MOH Madagascar’), managerial competences and supplies management (‘MOH Uganda’, ‘PSI Cameroon CIDA’, ‘PSI Malawi CIDA’) or gender based violence (‘PSI Malawi CIDA’).

Tools used in the training initiatives included full curriculums for clinical care, trainers–of–trainers manuals, facilitators’ guides, job aids, algorithms or lists of supplies. Two programmes explicitly reported that materials were based on WHO/UNICEF or MOH materials which were adapted to local situations (eg, ‘MC Sudan South’, ‘MOH Malawi’).

Supervision was designated or assimilated to several human resources management strategies (eg, managerial supervision, clinical supervision, mentorship). In some programmes, several cadres could be responsible for CHW supervision: for example, in ‘Concern Burundi’ supervisors included Concern staff, District Health Teams and health centre staff; in ‘MC Uganda’ included health centres’ staff, Community Development Officers, Health Assistants or Health Inspectors; senior CHW, environmental officer or community nurses (‘MOH Malawi’);community based Health Area focal points and Animateurs District CCM focal points (‘PSI Cameroon); senior CHW, Environmental Health Officers or Health Facility Staff (mentors) (‘PSI Malawi’); senior CHW (routine supervision) and health centres’ clinical staff (clinical mentors) (‘Save Malawi’)

In other programmes, supervision was assigned to a single cadre: a community health in–charge (‘Concern Rwanda KabehoMwana’), a senior CHW (‘CORE Malawi’), health centre staff (‘IRC Sierra Leone’), programme officers (‘MC South Sudan’), nursing holders of health areas (‘PSI DRC’), or even community members (‘MOH Uganda’); centre technical director (‘PSI Mali’); field leader of township (‘PSI Myanmar’); CHW supervisors (‘PSI South Sudan’, ‘Save South Sudan’); chief nurse (or medical technician) (‘Save Mozambique’, ‘USAID BASICS DRC’). No information data was available regarding the gender mix of supervisors.

Supervisors undertook a specific training, which ranged from two to nine days, in the six programmes where this information was available. Tools used included guidelines, checklists and training manuals.

The supervisors: CHW ratios varied: 1:2 (‘PSI Malawi’), 1:6 (‘PSI DRC’), 1:6 to 7 (‘Save Mozambique’), 1:8 to 16 (‘IRC Sierra Leone’), 1:10 (‘PSI Cameroon’), 1:11 (‘Save Malawi’) or 1:18 (‘Save South Sudan’).

The frequency of supervisory visits ranged from once a year to three times a month (‘PSI South Sudan’); although in some cases there are reports of CHW not having received a single supervisory visit (eg, ‘MOH Madagascar’). Meetings were also mentioned as supervision–like strategies in seven programmes (37% of the 19 programmes with this information available) (eg, ‘MOH Ethiopia’ with biannual meetings; ‘PSI DRC’ monthly monitoring meetings).

Supervision activities could include any mix of the following areas of work: clinical skills, submission of reports, analysis of reports and feedback, medical supplies, logistics, site management, relations with the community, recommendations or corrective actions.

Interestingly, there were programmes where CHW were working within a more or less formal network of CHW and other providers. For example, teams and team–work was heavily emphasised in ‘Save Zambia’; ‘Care Group Volunteers’ were reported by ‘Concern Burundi’; and peer support groups based on the Care Group model were implemented by ‘Concern Rwanda KabehoMwana’.

Several tools were identified across the documents, sometimes clearly highlighted in programmes reports and some other times identified by the reviewers as potentially innovative or particularly important programme components. A total of 114 tools have been identified across the whole set of programmes. In summary, they included equipment (eg, a backpack and storage box, a drug calculator, supplies, as complements to CHW activities and to support motivation as well); guides (describing procedures or tasks, such as clinical tasks, assessments or supervision); job aids (eg, home–based management; peer–support groups; case management; counselling cards, mother reminder cards); templates for reporting (eg, register and referral forms; CCM register; CCM supervision form; follow–up visit form; medication stock management form); communication tools (eg, home and community boards; flip charts). Other tools included an integrated analogue and digital mobile phone application for real–time stock tracking and reporting or an integrated toolkit map to facilitate the planning of activities within the catchment area of CHW.

### Quality of care

Eventually all programmes implemented IMCI care protocols in one way or another, which served as an overreaching framework for a number of activities with the main components being guidelines, expansion and training of CHW, supervision and often supplies.

Programmes were not uniform in their underlying ‘quality of care’ concept or framework, which was in most cases implicit. Therefore, ‘quality of care’ was approached under different perspectives and dimensions of care across programmes. We extracted information on a limited number of outcomes related to access (ie, utilisation, coverage), appropriateness of care (eg, adherence to guidelines) and health outcomes. Programmes reported very different outcomes and there was no full consistency in measurements and reporting approaches.

As shown in **Table 5**, we extracted and grouped reports on outcomes, selecting those that seemed to be better related to quality of care indicators and better reported. 43% were categorised as qualitative (‘+’), 30% as quantitative (‘++’) and 26% as quantitative with some estimation of statistical significance.

**Table 5:** Selected effects of programmes on quality of care related outcomes*

Programme	Access, quality of care, health outcomes	Quality of evidence†
Backpack	Optimize access and service efficiency; increased community trust thanks to better communication; reduced error rates thanks to improved tools and higher guidance. Better treatment thanks to enhanced guidance and real–time support (source: project statement); reduced stock outs.	+
Concern Rwanda‡ – Home Based Management Malaria Programme	Increase access and use of prompt treatment for presumed malaria (20% [CI 13% to 23%] to 43% [CI 35 to 51%]); increase access to zinc for diarrhoea (5% [CI 2% to 8%] to 22% [CI 15% to 30%]); more practice of giving increased liquids for diarrhoea (36 [CI 30% to 42%] to 57% [48% to 66%]); increase vitamin A coverage (66% [CI 61% to 71%] to 86% [78% to 94%]); increase practice of hand–washing with soap on key occasions (2% [CI 1% to 4%] to 19% [CI 11% to 26%])§. Notable improvements in treatment–seeking between 2005 and 2010 (greater in KabehoMwana districts). Treatment seeking from any provider for all three conditions combined increased from 16% to 46% in the KM districts vs 26% to 40% in non– KabehoMwana districts. Other indicators shown differences: soap availability, vitamin A supplementation, diarrhoea management, respiratory disease management.#	+++
	In most Health Centres assessed, reported malaria cases decreased during the peak malaria season in the year after implementation of HBM, compared to the year before.	++
CORE – Cameroon	Changes from baseline in the percentage of sick children correctly assessed and managed for danger signs (10.5% to 33.9%) and specific diseases (for example diarrhoea: from 23% to 66.7%).Coverage of certain interventions (eg, vaccination). Mothers’ knowledge.	++
CORE – Malawi	Estimated 1114 lives were saved over the life of the project, 474 from malaria (applying the lives saved calculator to data). Estimated cost per life saved US$ 1200 (based on the project’s total budget).	++
	Mothers continued breastfeeding children even when pregnant; children and pregnant women were more likely to eat eggs, food high in protein and essential micronutrients. Care–seeking for childhood illness increased from 71% to 84%; childhood vaccinations increased from 69% to 96%; vitamin A dosing increased from 54% to 82%; exclusive breastfeeding jumped from 40% to 82%.	++
	Residents far less likely to use traditional healers; people stopped using bed nets for fishing; a significant number of traditional healers abandoned their practice and joined the program as volunteers, isolating and undermining the credibility of those who remained working as traditional healers.	+
IRC Sierra Leone	Care–seeking changes (2010 to 2013): overall (82.0% [CI 76.7% to 88.2%] to 72.4% [CI 62.6% to 80.5%]) and malaria /fever (57.4% [CI 49.7% to 64.9%] to 83.8% [CI 77.9% to 88.4%]); time delays reduced for diarrhoea but increased for pneumonia. First sources of health care in 2010 and 2013 for sick children (CHW52.0% to 52.9%), for children who died (governmental health facility CBD 52.9% to 24.9%; CBD37.7% to 30.8%). Treatments/child/y given by CBD (2010 vs 2013): malaria (0.56 to 1.37), diarrhoea (0.52 to 0.88), pneumonia (0.46 to 0.31). Appropriate treatment (2010 to 2013): malaria (54.7% to 80.4%), diarrhoea (33.1% to 53.7%), pneumonia (0.0% to 67.8%). Prevalence: malaria (46% to 36%), diarrhoea (5% to 7%), pneumonia (1% to 6%). Mortality 2 to 59 mo: statistically non–significant reduction, from 2010 to 2013.	+++
Living Goods Uganda	Better access to diagnostics. The results are consistent with a simple experience model where biomedical misconceptions decrease consumers’ ability to infer quality.	+
MC Uganda	Communication outcomes: sick child job aid is a trusted guide for both CHW and caregivers and appears to contribute to quality of care; interpersonal skills are the key drivers of caregivers’ satisfaction, impacting positively on the CHWs’ clinical skills.	+
MOH Madagascar	CHW referred to health facilities: 71.6% (69.9% to 73.3%) of children with severe illness or other indications; chose the appropriate life–saving treatment when it was needed only 53% (43.3% to 63.1%); chose RTDs when indicated only 55% of the time; assess contraindications for oral contraceptive use only 41% of encounters.	+++
MOH Malawi	Communities are using the sick child services.	+
PSI Cameroon	Reduction of mortality in one district but not in another one (from 96.8 to 86.7/1000 life birth); increased access to the poorest (52% among the poorest vs 35% among the less poor); for ACTs: 45% vs 33%. Improved quality of care.	+++
PSI Malawi CIDA	Slight reduction in stock–outs and slight increase in health seeking behaviour for diarrhoea, fast breathing and fever.	+
PSI Mali	Treatment target (80%) was exceeded (average 81% and 86% at the end of the period). 713 474 DALYs (8399 deaths averted).	++
	Mild to moderate improvements in appropriate treatments, positive care–giver feed–back.	+
PSI Madagascar	Trust of community members; although some are sceptical. Statements on supply management and sales.	+
PSI Myanmar	Access to RDT. Increase of ORS use. Cost–effectiveness of ORS distribution (US$ 431/DALY).	++
PSI Uganda	No evidence of changes in coverage; changes in case management comparable to national levels; may be stronger gains in children from less poor households.	+
Save Malawi	CHW were the main source of care in intervention areas (at baseline the source was the public sector); shifting care from public to CHW care; checking breathing with timer not systematic; non–statistical significant increase of appropriate treatments. Improved on equity in access.	+++
Save Mozambique	Health seeking for fever: higher in intervention areas for the formal sector (intervention clusters 83.2% (CI 76.3 to 90.0); comparison areas 66.3% (95% CI 57.8 to 74.9)); CWH were the main source; pneumonia: lower in intervention areas in the formal sector; diarrhoea: CHW main source in intervention areas; first–line antimalarials: CHW preferred in intervention areas and formal sector in comparison areas. Improve of early treatment for malaria and diarrhoea (significant) and pneumonia (hardly significant). 2/3 RDT and less used timer for breathing. Provision of correct drugs (80%). Increased knowledge by mothers in intervention areas.	+++
IRC Ethiopia	Some practices not followed (check for danger signs, correct assessments).	+
Sizika	100% completeness in electronic data upload; 98.44% of promptness of treatment; 57% of treated children; 70% of relay/CHW were supervised; 75.5% to 98.44% of early malaria treatments (depending on month).	++

CI –confidence interval, CIDA – Canadian International Development Agency, DALY – disability adjusted life years, DRC – Democratic Republic Congo, HBM – home based management of malaria, IRC – International Red Cross, MC – Malaria Consortium, MOH – Ministry of Health, ORS – oral rehydration salts, PSI – Population Services International, RDT – rapid diagnostic test

*Not all programme reported information on this area.

†Sources of evidence are qualitative data or opinions (+), quantitative methods described in the source documents (++), or findings are presented with some measure of statistical significance (+++).

‡Two similar documents sharing a common author and similar sources were available on the Peer Support Group topic specifically so the outcomes were captured from the most comprehensive document (PSG Review paper). Case study on Peer Support Group drawing from primary and secondary data collected as part of the KabehoMwana project final evaluation, prepared for the iCCM Symposium evidence review; consists of an adaptation of a longer paper describing experiences from the USAID-funded KabehoMwana project in Rwanda. Review paper on Peer Support Group combining participant observations from designers and implementers of the CHW PSG model, with project monitoring and routine monitoring data, findings from primary and secondary data collected as part of the KabehoMwana final evaluation; and other available studies in the grey literature.

§Final project external evaluation (2011) with a knowledge, practice and coverage survey, comparison with 2007 baseline. Interviews were conducted with 120 mothers of children 0 to 23 mo, and 395 mothers of children 0 to 59 months who had been sick in the last two weeks with at least one the following conditions: fever or malaria, diarrhoea, respiratory symptoms. In total, 120 villages were sampled, and 473 households were interviewed. Household selection was made according to an algorithm.

#Demographic and Health Surveys 2005 and 2010.

The synthesis of effects on quality of care suggested that there were positive effects for those strategies that involved policy change (‘CORE Cameroon’), organisational change (eg, C–IMCI framework ‘CORE Cameroon’), standardisation (‘Concern Rwanda’), integration with existing health care services and alignment with other programmes which may ease implementation and scaling up (‘CORE Malawi’, ‘PSI Mali’).

Quality changes seemed more remarkable in large multi–component programmes which included training of CHW, strengthened supervision and improvement of supply change management. Improvements in monitoring and evaluation procedures seemed to have had positive effects on utilisation rates (‘MOH Ethiopia’). Interestingly, access improved in most programmes, yet achievements were moderate in absolute terms or compared with formal health care (‘Save Malawi’). Geographic and effective access to care increased (‘Save Malawi’). A programme with a component of improvements of information transmission through mobile telephones seemed to have increased utilisation of CHW and more prompt management of illnesses.

Other strategies aimed at reinforcing the relations between CHW either with peers or supervisors. Peer–support groups provided a platform for more effective human resources interventions (eg, supervision, trust, accountability; ‘Concern Rwanda’); social franchising (‘PSI Myanmar’) seemed to strengthen networking of providers, alongside an increase in reputation of CHW. Micro–franchising seemed to achieve affordable improvements in the availability of good quality medical products (‘Living Goods Uganda’). Social franchising increased the availability of services at equal or lower costs than regular formal services and supplies with specific data on Oral Rehydration Salts (ORS) distribution (‘PSI Myanmar’). Equity (differentials in access from different economic strata) was reported in terms of access to CHW, Artemisinin–based Combination Therapy (ACT) and treatments of diarrhoea (‘PSI Cameroon’); social franchising also seemed to improve equity, focusing on the most vulnerable populations (‘PSI Myanmar’, ‘PSI Uganda’).Yet, at least one programme (‘Save Malawi’) could not find differences in accessing CHW according to wealth.

Effects on caregivers seemed positive, with increases in knowledge, changes in care seeking behaviour (‘PSI Malawi’, ‘PSI Mali’) and caregivers’ basic disease management (‘Concern Rwanda’); clients’ satisfaction with availability of medicines and care increased (‘PSI Cameroon’); and there were some indications that community ownership and accountability were strengthened.

Effects of iCCM interventions may not be sustained over time everywhere since there were examples of declining coverage of services (‘PSI Cameroon’) with time. Social franchising showed examples where coverage of services did not seem to increase (‘PSI Uganda’).

In contrast, some of the poor outcomes (eg, clinical management) were related to the shift of care seeking between different types of providers; for example, from formal governmental services to CHW community based care. Training large numbers of CHW led to the reduction in the use of traditional healers, although this was seen as a positive effect of the programme (‘CORE Malawi’). Introduction of an iCCM programme in an area where care seeking appeared generally high resulted in shifting of care from government health centres, private health facilities and shops to village health clinics.

Although the aims of this review did not include reporting on mortality, it is worth noting that reductions in mortality were occasionally reported with findings suggesting reductions in some geographical areas but not in others, within the same programme (‘PSI Cameroon’), or not statistically significant reductions (‘IRC Sierra Leone’).

## DISCUSSION

We have reviewed 29 iCCM programmes in Sub–Saharan Africa and Myanmar. All programmes were based on iCCM guidelines and principles implemented by CHW, although the way programmes were implemented varied greatly. This review had some limitations: it is likely that more programmatic or research information could have been found with more time and resources; information was typically retrieved from evaluation studies or programmatic documents rather than experimental, controlled research studies providing moderate to low quality evidence; finally, reporting bias could not be excluded since only reports provided by a selection of stakeholders were included in this review. While programmatic information provides invaluable evidence on processes, the lack of more robust evidence on the effects of the programmes precludes any attempt to relate processes with outcomes. Findings from this review are not and cannot be representative of iCCM programmes and cannot be extrapolated to any particular setting. However, they are meant to help to draw lessons from those programmes proposed included in this review.

iCCM was defined by its objectives by WHO/UNICEF in 2012: “to train, supply and supervise front–line workers to treat children for both diarrhoea and pneumonia, as well as for malaria in malaria–affected countries, suing ORS and zinc, oral antibiotics, and artemisinin–based combination therapy (ACT) ... iCCM also enables community health workers to identify children with severe acute malnutrition through the assessment of mid–upper–arm circumference (MUAC)” [4]. This definition shares the objectives and resembles the old definition of selective primary health care (PHC) (1979): “a circumscribed number of diseases are selected for prevention in a clearly defined population ... The principal recipients of care would be children up to three years old and women in the childbearing years. The care provided would be measles and diphtheria–pertussis–tetanus (DPT) vaccination for children over six months old, tetanus toxoid to all women of childbearing age, encouragement of long–term breast feeding, provision of chloroquine for episodes of fever in children under three years old in areas where malaria is prevalent and, finally, oral rehydration packets and instruction” [10].Similarities between both approaches suggest that iCCM is not an entirely new strategy, but rather it shares and it may be inspired by key features of selective PHC.

In this article we focused on the description of programmes and quality of care issues. iCCM programmes are composed by a mix of multiple interventions or strategies; namely, disease portfolios, CHW arrangements, clinical skills, supplies, referral systems, training, supervision, community support and policy changes. These components are implemented in different combinations and intensities depending on the country or setting where programmes operate, donors’ preferences and country health related policies, among other factors. It was appealing to us that, in fact, the term ‘iCCM’ embraced a large plethora of very different programmes which may have limited commonalities between them in some cases. Most studies, trials or evaluations clearly deal with key factors affecting the performance of CHW, the quality of care they provide and, eventually, clinical outcomes. These factors included supplies, CHW supervision, training, quality of care and retention of CHW [11], among others.

A contribution of this review of iCCM programmes has been to systematically identify and present innovative or promising approaches; such us: integration with other programmes to boost effectiveness; integration with the private, public and traditional sectors, coordination with stakeholders to align resources and expertise in different areas of work. Stock management and availability of medicines seemed to be key in several programmes in effectively supporting CHW activities and ensuring credibility.

Measuring quality of care in the context of CCM is challenging and different approaches and measurement methods may lead to different descriptions of the same situations [12]. The number of quality of care indicators is very large and their types extremely varied, for example from the availability of inputs to the achievement of outputs and outcomes, from knowledge to health status outcomes, and the perspectives of supply– and demand–side. Therefore, one could argue that it is easy to find examples of positive effects when a large number of indicators are measured, as was the case in the programmes we have reviewed. As in other reviews, care seeking behaviour and utilisation of treatments tended to show positive, albeit variable, effects across the different programmes [5]. Not so often, examples of no effect were found; even less frequently, negative effects were identified (reporting bias of positive outcomes could not be investigated nor ruled out). Interestingly, the shift of utilisation from traditional healers to CHW was reported as a positive outcome, when actually this might not necessarily be seen as a desirable outcome (‘Core Malawi’).

We acknowledge, that quality of care is a means to achieve better health related outcomes, such as morbidity and mortality [13]. Only limited evidence on morbidity and mortality has been included in the documents of the programmes we have scrutinised. Interestingly, there are variations in the effects of community based management across conditions. For example, a review of the evidence on the effects of community based management of pneumonia in Africa, which included published studies in English or French (excluding non–published reports), using any primary study design, could not find evidence of impact on morbidity and mortality and raised several implementation concerns related to CHW capacity to manage pneumonia [6]; although more promising findings were reported in another review which included studies from Asia [5]. More positive findings were reported in a systematic review of experimental or quasi–experimental studies in Sub–Saharan Africa on the effects of CCM on malaria outcomes [14]; for example, significant reduction of malaria deaths in Ethiopia and Uganda, although no effects were observed on other clinical outcomes (eg, hospitalisations, anaemia). Often, though, the quality of evidence is not optimal [15].An additional issue to consider is the role of programme characteristics (ie, the specific implementation approaches) and the context. The scope of our review does not allow to drawing conclusions about differences on iCCM performance in different geographical areas (eg, Africa or Asia); however, where evidence exists, these differences have not stood out [5].

## CONCLUSIONS

We attempted to provide some insights on the effects of iCCM intervention in quality of care indicators, despite the fact that scoping reviews do not aim at establishing effects of interventions. Large, multi–faceted, integrated iCCM programmes, with strong components of training, supervision, which included additional support of equipment and supplies, provided examples of improvements in selected quality of care outcomes. However, examples of modest, null and somehow adverse effects were also shown.

We could not establish which mix of interventions or strategies (eg, supervision, training and incentives) produced which effects on quality of care. Evidence on the main components exists; for example, on lay health workers [16], supervision [17], training or job aids [18]; but not on the mix of those interventions which lead to better outcomes and under which conditions. We are afraid that this is also the case for the reviews and studies we have recently accessed. Inevitably, the effects of innovative approaches (eg, networking between CHW peers, mentorship), which were used and seemed promising in some cases, remained diluted in the body of low quality evidence that could be extracted from programmatic documents. The lack of good quality evidence is not only a concern for the international health community, but also for policy makers [19] who may not recognise the value of an approach which may not have been robustly evaluated and reported. May be evidence on the strengths and limitations of selective PHC could have also informed more recent initiatives to implement iCCM.

In the absence of good quality evidence, research evidence has to be produced [20] and, in the meantime, good quality global guidance on what iCMM ‘formula(s)’ are more promising under different circumstances, needs to be elaborated with tools to adapt it to local settings [21]. A research agenda, and eventually guidance developers, would benefit from a series of actions spearheaded by the iCCM task force; namely: (i) standardisation of iCCM concepts, strategies and tools; (ii) establishment and reinforcement of evaluation methodological standards, including protocols for selecting and reporting on primary outcomes, harms and costs; (iii) mapping existing research to avoid duplications and search synergies, emphasising pragmatic research of integral approaches rather than for individual diseases; (iv) establishing a few manageable priority research areas; (v) creating an open, structured, transparent and comprehensive online platform to share evidence on iCCM with quality assessments of the evidence presented.
